# Extreme size mismatch: bronchus compression by an oversized donor heart in small children

**DOI:** 10.1016/j.heliyon.2022.e11095

**Published:** 2022-10-15

**Authors:** Hsun-Yi Fu, Heng-Wen Chou, Yi-Chia Wang, Nai-Kuan Chou, Yih-Sharng Chen

**Affiliations:** a Department of Cardiac Surgery, National Taiwan University Hospital Hsin-Chu Branch, Hsin-Chu, Taiwan; b Department of Cardiac Surgery, National Taiwan University Hospital, School of Medicine, National Taiwan University, Taipei, Taiwan; c Department of Anesthesiology, National Taiwan University Hospital, Taipei, Taiwan

**Keywords:** Case report, Heart transplant, Pediatric, Size mismatch

## Abstract

Studies have suggested that a more liberal criterion of donor–recipient weight ratio (DRWR) is associated with superior waitlist survival without compromising posttransplant outcomes in selected critically ill patients. Successful transplantation of an extremely oversized donor heart into a small recipient is herein described. A 2-year-old girl accepted a size-mismatched adult donor heart offer (DRWR of 4.4) due to frequent complications with a left ventricular assist device. During the immediate postoperative period, spatial constraints within the thoracic cavity compromised graft function. Computed tomography revealed severe compression of the left bronchus due to the oversized allograft with lobar collapse of the left lung. With temporary extracorporeal membrane oxygenation support, graft function improved within 1 month after transplantation. Subsequent adaptive size remodeling of the transplanted heart with concomitant left bronchus re-expansion was observed within 6 months after transplantation. Despite a complicated posttransplant recovery, the patient was discharged home with minimal respiratory sequelae. Our report describes an alternative strategy for managing early morbidities related to an oversized graft and supports extending the criteria of size matching in pediatric heart transplantations.

## 1 Introduction

The waitlist mortality of pediatric heart candidates is among the highest for all solid organ transplantation, even in the era of ventricular assist devices (VADs) [1]. Accepting marginal donors is one of the urgent strategies to decrease waiting time and to maximize organ utilization [2]. In recent decades, the donor discard rate has decreased; however, it remains at between 34% and 45%, and more than 30% of potential donor hearts are refused due to size mismatch [1, 3]. While expert consensus recommends a donor-recipient weight ratio (DRWR) of 0.6–3.0, studies have shown that a more liberal DRWR criterion is associated with lower waitlist mortality, more offers, and a higher rate of transplant, without compromising posttransplant survival [1, 4]. We report a case of pediatric heart transplantation with extreme size mismatch.

## 2 Case presentation

A 2-year-old girl with dilated cardiomyopathy received an urgent paracorporeal continuous-flow LVAD implantation in INTERMACS (Interagency Registry for Mechanically Assisted Circulatory Support) profile 1. Organ recovery following VAD implantation was encouraging, but her ambulatory rehabilitation was complicated by repeated episodes of embolic stroke due to pump thrombosis, which necessitated frequent pump exchange. Accordingly, we accepted an offer from a size-mismatched donor. The donor was a 36-year-old female victim of a traffic accident. The DRWR was 4.4 (53 kg–12 kg) and the donor-recipient height ratio was 2.0 (166 cm–82 cm). The maximal transverse diameter of the donor heart was 11.2 cm, and the maximal transverse diameter of the left hemithorax in the recipient was 9.4 cm, as measured using computed tomography (CT). Before implantation, the bilateral pleura were opened widely to accommodate the oversized allograft within the chest. The biatrial anastomosis technique was used. The donor's aorta was anastomosed to the lesser curvature of the aortic arch of the recipient. After operation, the recipient was placed on central venoarterial extracorporeal membrane oxygenation (10.13039/100000738VA ECMO) support for suspected primary graft dysfunction (PGD) and left bronchus compression.

**Figure 1:**
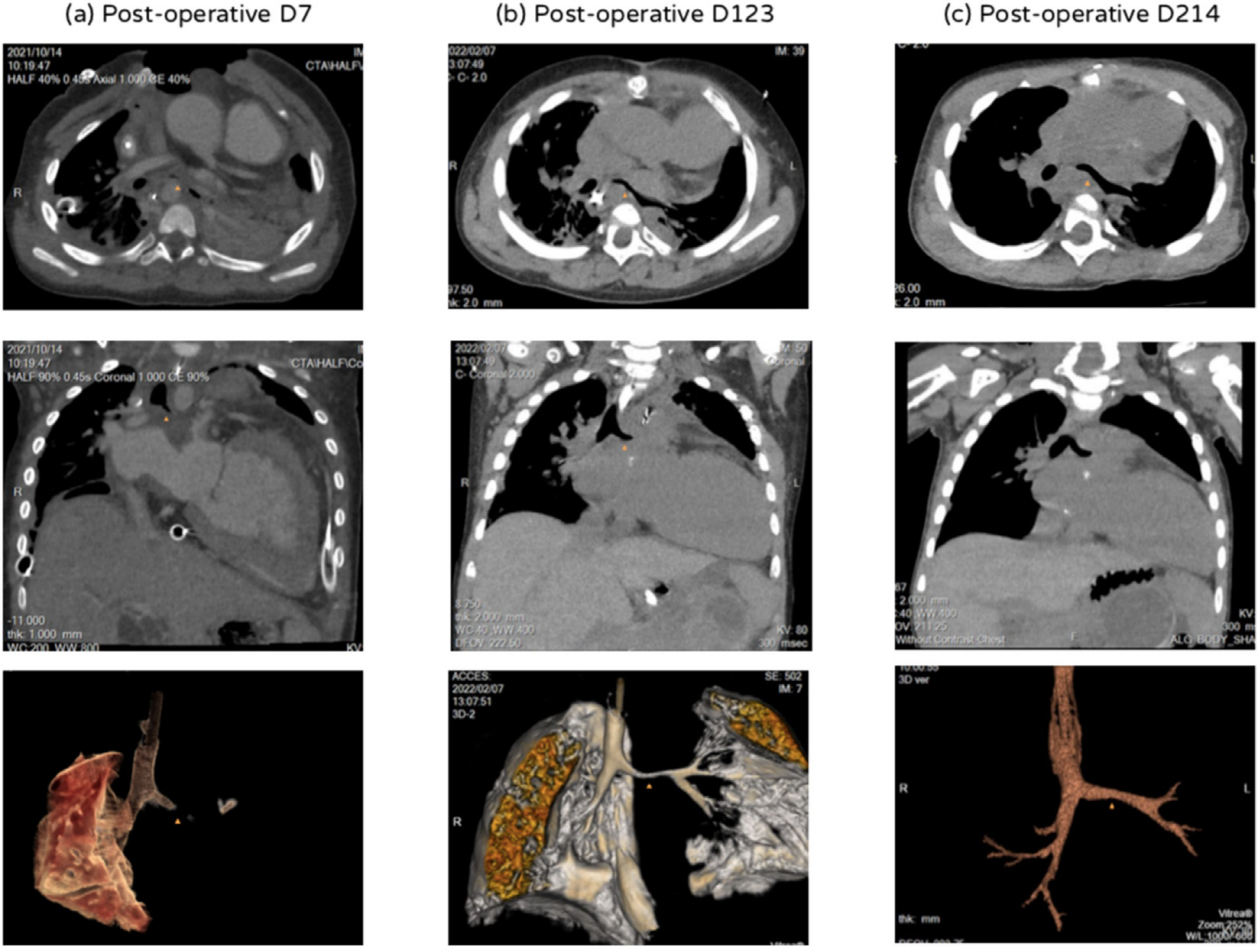
Computed tomography (CT) with image reconstruction (a, left panel) CT on POD 7 showed that the heart deviated the tracheobronchial tree to the right (upper), compressed left bronchus (lower), and occupied almost the entire left hemithorax (middle) (b, middle panel) CT on POD 123 showed a decrease in the size of the cardiac allograft (upper), relieved compression of the left bronchus (middle) and improved expansion of left lung (lower) (c, right panel) CT on POD 214 showed further size remodeling of the cardiac allograft (upper and middle) and only mild residual compression of the left bronchus (lower). ^▲^left bronchus. CT, computed tomography; POD, postoperative day.

**Figure 2:**
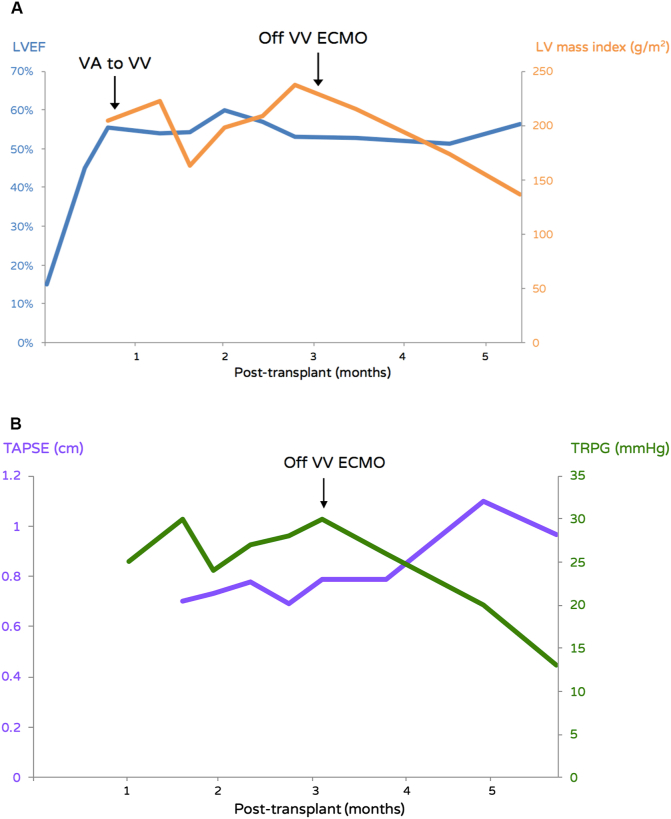
Echocardiography data. **A** LVEF (%) and left ventricular mass index (g/m^2^) after heart transplantation. **B** TAPSE (cm) and TRPG (mmHg) after heart transplantation. Arrows indicate the timing of change in ECMO configuration and ECMO decannulation. ECMO, extracorporeal membrane oxygenation; LVEF, left ventricular ejection fraction; VA, venoarterial; VV, venovenous; TAPSE, tricuspid annular plane systolic excursion; TRPG, tricuspid regurgitation pressure gradient.

In the first week after transplantation, bradycardia following CO_2_ retention was frequently noted while weaning from VA ECMO. CT revealed the heart deviated the tracheobronchial tree to the right, compressed left bronchus, and occupied almost the whole left hemithorax (Figure 1). Sternal closure was performed on post-operative day (POD) 10 with peripheral ECMO support. The pericardium was reconstructed with extended artificial patch coverage (Gore-Tex Soft Tissue Patch) to prevent possible sternal-cardiac and lung-cardiac adhesions, and the sternum was reapproximated using interrupted simple wires. Graft function had sufficiently improved by POD 29; thus VA ECMO was converted to venovenous (VV) ECMO. Tracheostomy was performed due to anticipated prolonged mechanical ventilation. Ventilator-associated pneumonia once complicated the recovery of pulmonary function, hence we arranged periodic bronchoscopic examination to monitor the collapse of the left bronchus and to improve airway clearance. Despite increased pulmonary vascular resistance, no evidence of rejection or neopulmonary stenosis was detected. VV ECMO was decannulated successfully on POD 96. Further adaptive remodeling in heart size and improved right ventricular (RV) function were observed in the following course (Figures 2A and 2B). The patient was transferred to the general ward on POD 136. A rehabilitation program and a home ventilator care plan were developed. Unassisted breathing through a tracheostomy collar was well tolerated, and a home ventilator (Resmed Astral) for use during sleep was connected. The patient resumed oral feeding and was able to ambulate with assistance; she was discharged home on POD 237. No hospital-acquired infections, including bloodstream, surgical site, or urinary tract infections were detected before discharge. Informed consent from the patient's parents was obtained for the publication of this article.

## 3 Discussion

Although evidence suggests that oversized cardiac allografts undergo size adaptation to accommodate the reduced requirements in small recipients within a few months after transplantation, a disproportionately large graft might lead to “big heart” hyperperfusion syndrome and compression of intrathoracic organs [5]. In our case, to prevent postoperative supravalvular neoaortic stenosis or neopulmonary stenosis, anastomosis modification without changing the orientation or downsizing the structure of the cardiac allograft was performed. However, spatial constraints of intrathoracic organs severely compromised postoperative heart and lung function. With successful ECMO support, serial postoperative 10.13039/100004811CT had clearly demonstrated the process of adaptive size remodeling of an extremely oversized donor heart, with the associated resolution of airway compression within 6 months after transplantation, which was rarely published in the field of pediatric heart transplant (Figure 1). Posttransplant complications from oversized donor hearts, including graft dysfunction, hypertension, bronchial compression, and respiratory complications are discussed below.

## 4 Primary graft dysfunction

Compared to the well-recognized risks of PGD from undersized donor hearts, the association between PGD and oversized donor hearts remains elusive [6, 7]. In the immediate postoperative period, the thoracic cavity in small recipients might constrain the oversized heart graft. Normal systolic function but compromised diastolic function of the ventricle with increased stiffness has been frequently observed in these oversized grafts [8, 9]. Additionally, although oversized allografts are expected to generate more pressure to overcome the increased pulmonary vascular resistance in recipients with complex congenital heart disease, there seems to be a trend toward an increased frequency of RV failure and the need for postoperative ECMO among patients with a higher ratio of DRWR [10]. In our case, left lung collapse could have further exacerbated RV dysfunction necessitating prolonged ECMO support.

## 5 Big heart syndrome

In pediatric heart transplantation, the so-called “big heart” or hyperperfusion syndrome caused by a disproportionately large donor heart is a well-recognized short-term complication of oversized grafts [11]. A vigorous, large graft may generate excessive cardiac output in a recipient who has previously endured a low-flow state until the completion of adaptive remodeling in the transplanted heart. Such an acute rise in cerebral blood flow with reactive vasoconstriction can lead to cerebral edema or ischemia manifesting as seizures, headache, or coma in the first few days after transplantation. Visceral and renal vasospasm due to systemic hyperperfusion have also been described [8]. Even though an oversized graft is believed to contribute to the development of posttransplant hypertension in most pediatric recipients, the incidence of long-term hypertension is not related to the initial level of donor-recipient weight mismatch [10]. No seizures, new neurological deficits, or acute kidney injury requiring renal replacement therapy occurred in our recipient before discharge. Her blood pressure was controlled within the normal physiological range with the use of small doses of a calcium channel blocker.

## 6 Pulmonary atelectasis and delayed sternal closure

The spatial constraints in small children could increase the risk of compression of other intrathoracic organs by oversized donor hearts. Recipients of hearts from donors of significantly larger size experienced more transient pulmonary atelectasis than recipients of hearts from more closely size-matched donors. However, studies have reported that clinical impairment of lung function is less likely to occur as a result of an oversized donor heart [12]. Elective delayed sternal closure until resolution of tissue edema, optimization of volume status, and stabilization of hemodynamics is a useful strategy for minimizing the risk of mediastinal compression during the early postoperative period, with a very low risk of morbidities, including sternal wound infection and mediastinitis [8]. To our knowledge, severe postoperative airway compression due to an oversized donor heart requiring VV ECMO has never been reported. The long-term prognosis of respiratory complications needs further investigation.

## 7 Conclusion

We presented a successful transplant of an extremely oversized donor heart into a small child following initial struggles with PGD and left bronchus compression. Remodeling of the oversized donor heart was clearly demonstrated by serial CT. Our report described an alternative strategy for managing early morbidities from oversized grafts and supports extending the criteria of size matching in pediatric heart transplants.

## Declarations

### Author contribution statement

All authors listed have significantly contributed to the investigation, development and writing of this article.

### Funding statement

This research did not receive any specific grant from funding agencies in the public, commercial, or not-for-profit sectors.

### Data availability statement

Data will be made available on request.

### Declaration of interest's statement

The authors declare no conflict of interest.

### Additional information

No additional information is available for this paper.
